# Fungal Farming in a Non-Social Beetle

**DOI:** 10.1371/journal.pone.0041893

**Published:** 2012-07-27

**Authors:** Wataru Toki, Masahiko Tanahashi, Katsumi Togashi, Takema Fukatsu

**Affiliations:** 1 Department of Forest Science, Graduate School of Agricultural and Life Sciences, The University of Tokyo, Bunkyo, Tokyo, Japan; 2 National Institute of Advanced Industrial Science and Technology (AIST), Tsukuba, Japan; University of Osnabrueck, Germany

## Abstract

Culturing of microbes for food production, called cultivation mutualism, has been well-documented from eusocial and subsocial insects such as ants, termites and ambrosia beetles, but poorly described from solitary, non-social insects. Here we report a fungal farming in a non-social lizard beetle *Doubledaya bucculenta* (Coleoptera: Erotylidae: Languriinae), which entails development of a special female structure for fungal storage/inoculation, so-called mycangium, and also obligate dependence of the insect on the fungal associate. Adult females of *D. bucculenta* bore a hole on a recently-dead bamboo culm with their specialized mandibles, lay an egg into the internode cavity, and plug the hole with bamboo fibres. We found that the inner wall of the bamboo internode harboring a larva is always covered with a white fungal layer. A specific Saccharomycetes yeast, *Wickerhamomyces anomalus* ( = *Pichia anomala*), was consistently isolated from the inner wall of the bamboo internodes and also from the body surface of the larvae. Histological examination of the ovipositor of adult females revealed an exoskeletal pocket on the eighth abdominal segment. The putative mycangium contained yeast cells, and *W. anomalus* was repeatedly detected from the symbiotic organ. When first instar larvae were placed on culture media inoculated with *W. anomalus*, they grew and developed normally to adulthood. By contrast, first instar larvae placed on either sterile culture media or autoclaved strips of bamboo inner wall exhibited arrested growth at the second instar, and addition of *W. anomalus* to the media resumed growth and development of the larvae. These results strongly suggest a mutualistic nature of the *D. bucculenta*-*W. anomalus* association with morphological specialization and physiological dependence. Based on these results, we compare the fungal farming of *D. bucculenta* with those of social and subsocial insects, and discuss ecological factors relevant to the evolution of fungal farming in a non-social insect.

## Introduction

Cultivation mutualism is a form of organism-organism symbiotic associations wherein an organism (normally called host) cultures another organism (often called symbiont or crop) as food source. Eusocial insects like ants and termites and subsocial insects like ambrosia beetles exhibit sophisticated forms of cultivation mutualism, which may be comparable to human agriculture in many aspects [1], [2]. These insects inoculate their specific fungal associates onto appropriate substrates, engineer the environmental conditions for their optimal growth, defend them against pests/parasites/pathogens by monitoring, sequestration and/or antibiotic application, harvest and consume them as food, and are obligatorily dependent on them [1]–[6]. Such specialized cultivation mutualisms are not found in non-social organisms. Only primitive forms of cultivation mutualism have been reported from non-social organisms like a marine snail with a fungus [7] and a damselfish with an alga [8], and also from a slime mold with food bacteria [9]. Among non-social insects, several cases of putative cultivation mutualism have been described [10], [11]. A well-documented case is the gall midges of the tribes Lasiopterini and Asphondyliini (Diptera: Cecidomyiidae). These insects carry their specific fungal associate and inoculate the fungus to their host plant upon oviposition. The fungus is suspected to be involved in the gall formation, proliferates in the inner space of the gall, and is consumed by the insect larvae [11]. Another well-documented case is the leaf-rolling weevils of the genus *Euops* [12]–[14]. External structures specialized for harboring fungal associates, designated as fungal pockets or mycangia, have been identified not only in social ants and ambrosia beetles with cultivation mutualism [15], [16] but also in various non-social insects such as lymexylid beetles, wood wasps, gall midges, leaf-rolling weevils, stag beetles and others [10], [12], [17]–[20]. In these non-social insects, however, biological aspects of the mycangium-associated microbes, in particular their physiological roles for their hosts, have been poorly investigated.

**Figure 1:**
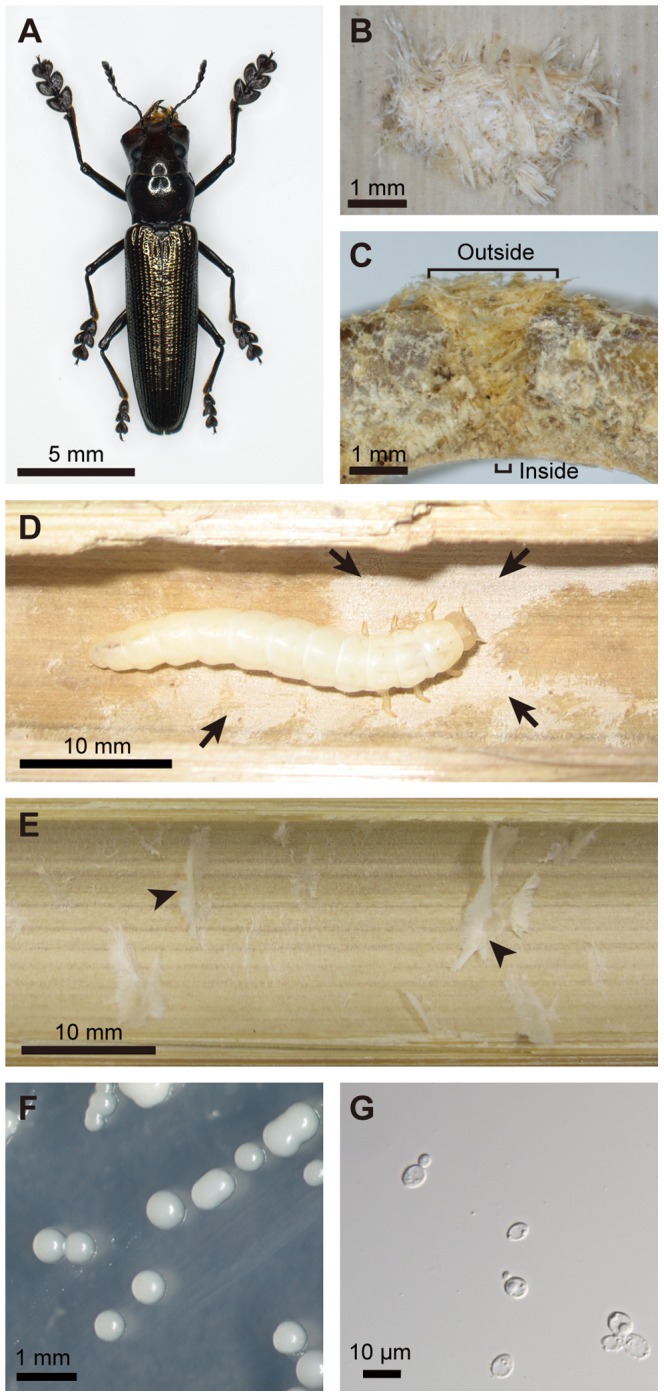
The lizard beetle *Doubledaya bucculenta*, the host bamboo *Pleioblastus simonii*, and the associated yeast *Wickerhamomyces anomalus*. (A) An adult female of *D. bucculenta*. (B) An oviposition mark of *D. bucculenta*. An outside view of an oviposited dead bamboo culm is shown. (C) A cross section of an oviposited dead bamboo culm. The oviposition hole is wider outside and narrower inside. (D) A larva of *D. bucculenta* feeding on fungal layer on the inner surface of the bamboo internode. Arrows indicate the fungal layer. (E) The inner wall of the bamboo internode without oviposition mark. Arrowheads indicate pith tissues of the bamboo. (F) Yeast colonies isolated from *D. bucculenta* on a potato dextrose agar plate. (G) A light microscopic image of budding yeast cells obtained from *D. bucculenta*.

The lizard beetles of the tribe Languriini (Coleoptera: Erotylidae: Languriinae) constitute a moderately diversified taxon, consisting of more than 1000 species worldwide, and have been reported to be phytophagous [21]–[23]. Adult females of the lizard beetle *Doubledaya bucculenta*, which is endemic to Japan, have a large asymmetric head with enlarged mandibles and elongated forelegs (Fig. 1A). In spring, they excavate a small hole on a recently-dead culm of *Pleioblastus* and *Semiarundinaria* bamboos, lay an egg into the cavity of the bamboo internode via the hole, and plug the hole with bamboo fibres (Fig. 1B and C) [24]–[27]. Within the bamboo internode, a single larva of *D. bucculenta* develops and pupates, overwinters either as larva or adult, and an adult insect emerges by biting out the bamboo wall in spring. To our knowledge, there has been no report on beetles of the subfamily Languriinae associated with symbiotic microorganisms [28]–[30].

In this study, we demonstrate that (i) the inner surface of the bamboo internode harboring a larva of *D. bucculenta* is always covered with a fungal layer, (ii) a specific fungus is consistently isolated from the inner wall and the larva, (iii) adult females of *D. bucculenta* possess an ovipositor-associated mycangium that contains the fungal cells, and (iv) the fungus is essential as food source for growth and development of the larvae of *D. bucculenta*, which provides a novel case of cultivation mutualism in this non-social insect.

## Materials and Methods

### Materials

Almost all insect samples of *D. bucculenta* and internode samples of the bamboo *Pleioblastus simonii* were collected at Kawaminami, Miyazaki Prefecture, Japan [32°9′N, 131°29′E]. In addition, a larval sample was collected at Shirosato, Ibaraki Prefecture, Japan [36°38′N, 140°34′E] on 24th June 2007, and an adult female was sampled at Takanabe, Miyazaki Prefecture, Japan [32°6′N, 131°31′E] in September 2009. The relatively limited sample size of the beetles was due to difficulty in collecting a large number of *D. bucculenta* [24]. No specific permits were required for the described field studies. The locations are not privately-owned or protected in any way. The field studies did not involve endangered or protected species.

### Microbial isolation

Seventeen bamboo internodes were collected on 4th December 2008 at Kawaminami, washed in running tap water, immersed in 70% ethanol and fire-sterilized. Then, each of the internodes was carefully cut open, and the inner wall was scraped with a tip of nichrome wire and transferred onto potato dextrose agar (PDA) (Difco, Detroit, MI, USA) plates containing no antibiotics. A larva collected from a dead *P. simonii* internode at Shirosato was placed on a 9 cm malt agar (Difco) plate containing no antibiotics, and allowed to walk for five minutes. Plates were incubated at 25°C until visible microbial colonies appeared. The microbial isolates were kept at 5°C during the experiments and stored in glycerol at −80°C for long-term maintenance.

### Rearing experiments

The above-mentioned fungal isolate from a larva of *D. bucculenta* was used for rearing experiments. The fungal cells were suspended in sterilized water, and 9 cm PDA plates were wiped with tissue paper immersed with the cell suspension (about 1 ml each) and incubated at 25°C for two days. Eggs of *D. bucculenta* were collected from dead *P. simonii* internodes on 5th and 7th May 2008 at Kawaminami, surface-sterilized in 99.5% ethanol for one minute and then in 70% ethanol for one minute, and placed on wet filter paper in a sterilized Petri dish at 25°C. Newly hatched larvae were randomly allocated to either of three experimental groups. In the first group, each of five larvae was placed singly on a sterile 9 cm PDA plate. In the second group, each of five larvae was placed singly on a 9 cm PDA plate on which the fungal strain had grown. The larvae were transferred to fresh fungus-growing PDA plates at intervals of 13–15 days to avoid depletion of the fungus on the plates. In the third group, each of five larvae was placed singly on an autoclaved strip of *P. simonii* internode (about 15.5 cm×2.5 cm in size) in a sterilized test tube (3.0 cm in diameter and 20 cm tall) with moistened cotton placed at the bottom. These insects were reared at 25°C in the dark, observed daily to record molting, and weighed aseptically at intervals of 12–22 days during the initial 70 days. Since it was difficult to find larval shed skins in the test tubes, the duration of each instar could not be perfectly recorded. For the third group, after rearing for 70 days, the fungal suspension (about 1 ml each) was added to the bamboo strip in the test tube, and the rearing was continued.

### Histology

Five adult males and five adult females were obtained from internodes of dead *P. simonii* culms at Kawaminami on 29th August 2009. These insects were dissected under a dissection microscope and carefully examined for any mycangial structures on the exoskeleton and internal organs. Dissected mycangia were fixed with 3% formalin in PBS, embedded in paraffin, processed into serial tissue sections, stained with periodic acid-Schiff reagent and haematoxylin, and observed under a light microscope as described [31].

### DNA sequencing and phylogenetic analysis

Fungal DNA samples were prepared using lyticase (Sigma-Aldrich, St. Louis, MO, USA) and Wizard Genomic DNA Purification Kit (Promega, Madison, WI, USA). The following primers were used for polymerase chain reaction (PCR): LS1 (5′ –AGT ACC CGC TGA ACT TAA G–3′) and NL4 (5′–GGT CCG TGT TTC AAG ACG G–3′) for D1/D2 domain of 26S rRNA gene; and 983 (5′–GCY CCY GGH CAY CGT GAY TTY AT–3′) and 2218 (5′–ATG ACA CCR ACR GCR ACR GTY TG–3′) for translation elongation factor-1α (EF-1α) gene [32]–[34]. PCR products were purified using Qiagen PCR Purification Kit (Qiagen, Hilden, Germany), and directly sequenced using ABI Prism BigDye Terminators (Applied Biosystems, Foster, CA, USA) and ABI PRISMR 3130xl Genetic Analyzer (Applied Biosystems). Nucleotide sequence data reported in this study have been deposited to the DNA Data Bank of Japan with accession numbers AB640725-AB640728, AB713902 and AB713903. Multiple alignments of the nucleotide sequences were generated using the program ClustalX 1.83 [35]. Molecular phylogenetic analyses were conducted by maximum parsimony method using the program PAUP 4.0b10 [36] with 1,000 bootstrap replicates, heuristic searches, and nearest neighbor interchange branch swapping.

## Results

### Observation of bamboo internodes harboring larvae of *D. bucculenta*

We collected more than 100 dead internodes of *P. simonii* that had an oviposition mark (Fig. 1B) at Kawaminami, Miyazaki, Japan. Many of them contained a larva of *D. bucculenta*, while the rest of them contained either an adult of *D. bucculenta* or no insect. In addition, we also sampled ten dead internodes of *P. simonii* with no oviposition mark. When they were cut open and inspected, a white layer always covered the inner surface of the larva-containing internodes with oviposition mark (Fig. 1D), whereas the white layer was not observed on the inner surface of the larva-absent internodes without oviposition mark (Fig. 1E). As for the internodes with oviposition mark that contained either an adult insect or no insect, the appearance of the inner surface was not uniform: some were clean while the others were dirtily contaminated, but none of them exhibited the typical white layer.

### Microbial isolation from bamboo internodes harboring larvae of *D. bucculenta*

For microbial isolation, we collected the following samples at Kawaminami, Miyazaki, Japan: five living internodes without oviposition mark from three living culms of *P. simonii*, all of which contained no insect; five dead internodes without oviposition mark from three dead culms of *P. simonii*, all of which contained no insect; and seven dead internodes with oviposition mark from three dead culms of *P. simonii*, of which five contained a larva of *D. bucculenta*, one contained an adult male of *D. bucculenta*, and one contained no insect. When the inner surface of each of the bamboo internodes was scraped and inoculated onto PDA plates, no microbial colonies were obtained from all the five living and five dead internodes without oviposition mark. By contrast, all the five dead internodes containing a larva consistently yielded a number of uniform white colonies on the plates (Fig. 1F). Light microscopic observation revealed that the colonies consisted of yeast-like unicellular fungal cells (Fig. 1G). From the dead internodes with oviposition mark that contained either an adult or no insect, not only the yeast colonies but also filamentous fungi were obtained.

We also collected a larva of *D. bucculenta* from a dead internode of *P. simonii* at Shirosato, Ibaraki, Japan. When the larva was allowed to walk on a malt agar plate, a number of yeast colonies of the same type appeared on the plate.

### Molecular characterization of the yeast isolates from *D. bucculenta*

**Figure 2:**
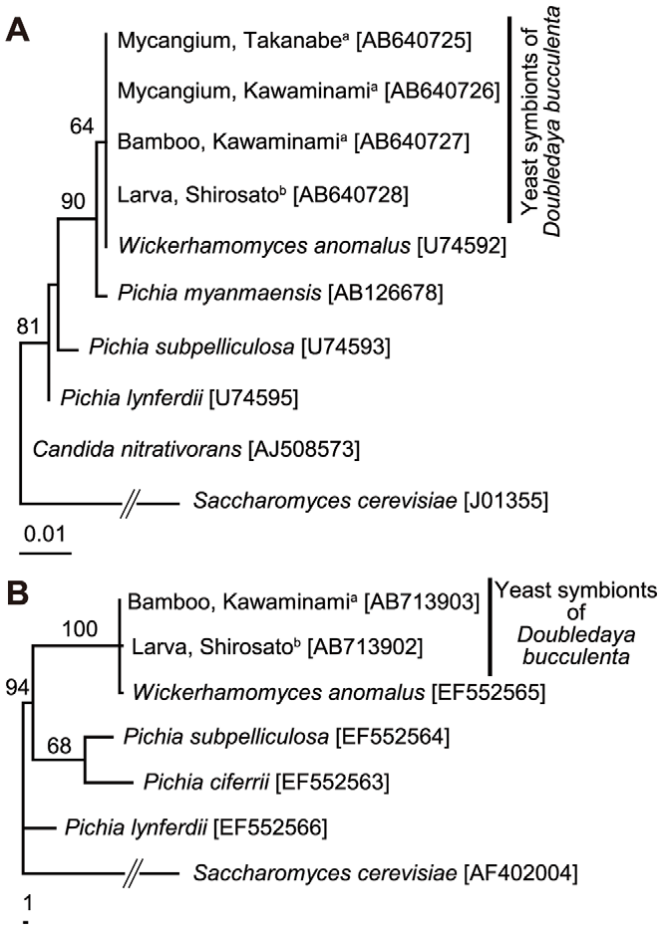
Phylogenetic placement of the yeast strains associated with *Doubledaya bucculenta*. (A) A maximum parsimony phylogeny inferred from 26S rRNA gene sequences (530 bps). (B) A maximum parsimony phylogeny inferred from EF-1α gene sequences (802 bps). Bootstrap values of 50% or higher are shown at the nodes. For each yeast strain obtained from *D. bucculenta*, isolation source and collection locality are indicated. Sequence accession numbers are in brackets. ^a^ Miyazaki Prefecture, Japan; ^b^ Ibaraki Prefecture, Japan.

A 0.5-kb fragment of 26S rRNA gene and a 0.8-kb fragment of EF-1α gene were amplified by PCR and sequenced from the yeast strains, one from a larva-containing bamboo internode collected at Kawaminami, Miyazaki, Japan, and the other from a larva collected at Shirosato, Ibaraki, Japan. The 530 bp sequences of 26S rRNA gene were identical between the yeast strains. The sequences were also identical to the sequence of *Wickerhamomyces anomalus* (formerly *Pichia anomala* [34]; accession number U74592 [37]) (Fig. 2A). Similarly, the 802 bp sequences of EF-1α gene were identical between the yeast strains except for one undetermined nucleotide site. The sequences were also identical to the sequence of *W. anomalus* (accession number EF552565 [34]) except for five nucleotide sites with ambiguous base reading (Fig. 2B).

### Discovery of a yeast-harboring mycangium associated with female ovipositor of *D. bucculenta*

**Figure 3:**
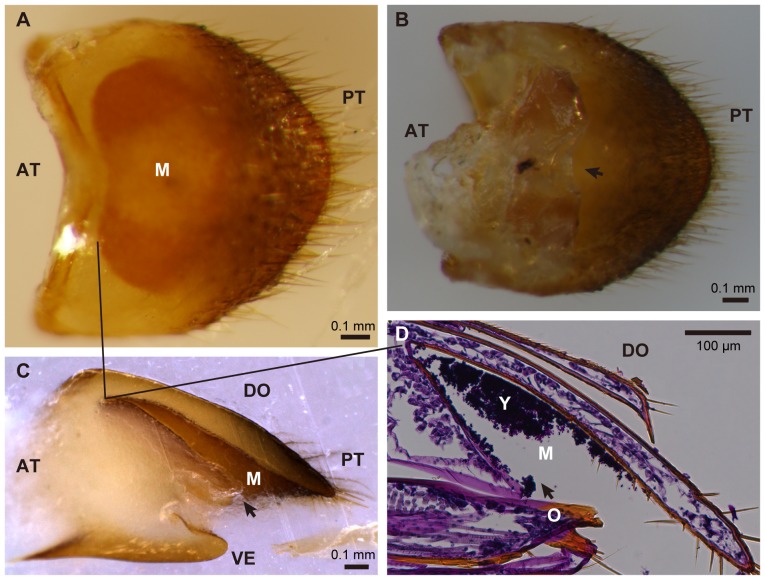
Mycangium associated with the ovipositor of adult females of *Doubledaya bucculenta*. (A) A dorsal view of the tergum of the eighth abdominal segment. (B) A ventral view of the eighth abdominal segment. (C) A lateral view of the eighth abdominal segment that was embedded in paraffin and cut longitudinally. (D) A longitudinal tissue section of the eighth abdominal segment, stained with periodic acid-Schiff reagent and hematoxylin. Arrows in (B), (C) and (D) indicate the opening of mycangium. Solid lines in (A), (C) and (D) correspond to the front edge of mycangial cavity. Abbreviations: M, mycangium; O, ovipositor; Y, yeast cells; AT, anterior; DO, dorsal; PT, posterior; VE, ventral.

In all five adult females of *D. bucculenta* collected at Kawaminami, Miyazaki, Japan, a yellowish exoskeletal pocket was present on the tergum of the eighth abdominal segment (Fig. 3A–C), while the structure was absent in adult males. Tissue sectioning identified yeast-like particles within the pocket (Fig. 3D). When the organ dissected from an adult female was subjected to DNA extraction, PCR and sequencing of the fungal 26S rRNA gene, the 530 bp nucleotide sequence obtained was identical to those of the already obtained yeast strains and also to that of *W. anomalus* (Fig. 2A). In addition, the same fungal sequence was obtained from the organ dissected from an adult female collected at a different locality, Takanabe, Miyazaki, Japan (Fig. 2A).

### Rearing experiments of *D. bucculenta* larvae in the presence and absence of the yeast

**Figure 4:**
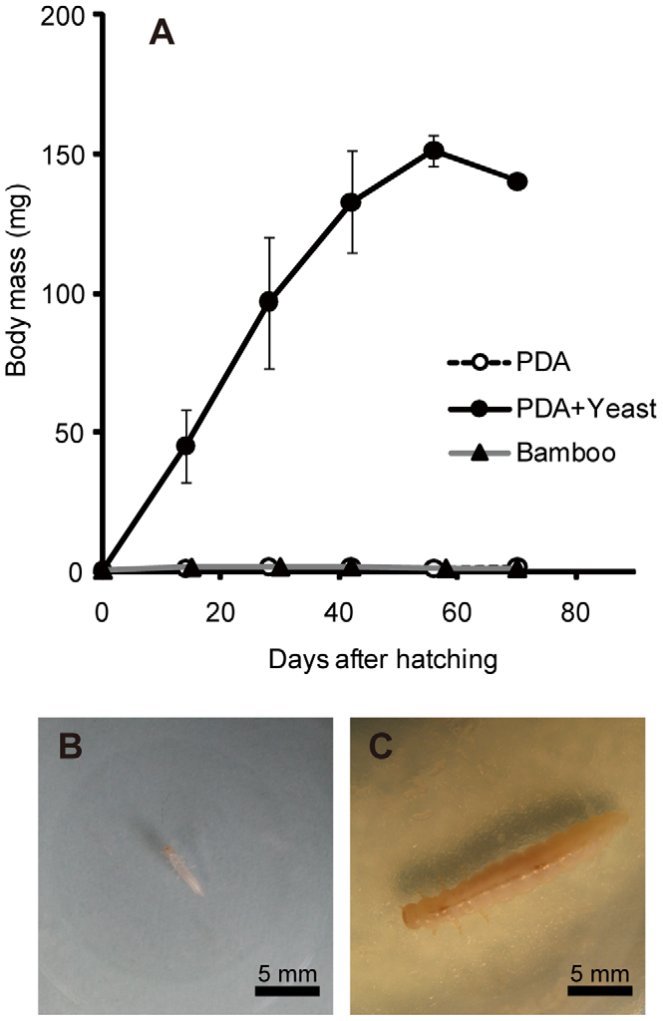
Effects of the yeast symbiont on larval growth of *Doubledaya bucculenta*. (A) Larval growth curves on sterile potato dextrose agar (PDA) plates (open circles), on PDA plates on which the yeast strain was fully grown (filled circles), and on sterilized bamboo strips (filled triangles). Means and standard deviations are shown. (B) A 14-day-old larva reared on a sterile PDA plate. (C) A 14-day-old larva reared on a yeast-inoculated PDA plate.

When five first instar larvae of *D. bucculenta* were individually reared on sterile PDA plates, four larvae managed to become second instar (mean and standard deviation of first instar period, 8.0±2.0 days, n = 4) but neither grew nor developed further (Fig. 4A and B). Among them, only a larva survived for 70 days after hatching, but it was very frail and died soon. A larva died in first instar.

By contrast, when five first instar larvae were individually reared on PDA plates on which the larva-derived yeast strain had fully grown, all five larvae promptly became second instar (mean and standard deviation of first instar period, 5.6±1.3 days, n = 5) and, strikingly, normally grew, pupated, and reached adulthood (Fig. 4A and C). The final instar differed among the larvae: fifth instar for two insects (larval period 40 and 54 days for one female and one male, respectively) and sixth instar for three insects (larval period 60, 58 and 73 days for one male and two females, respectively). The elytral length ranged from 10.7 to 11.6 mm for the two males and 10.9 to 11.2 mm for the three females. These values were within the range of the elytral lengths of field-collected *D. bucculenta* [26].

When five first instar larvae were individually reared on autoclaved strips of *P. simonii* internodes, four larvae managed to develop to second instar, while a larva died in first instar. Three of the larvae remained at second instar for 70 days after hatching, while a larva died during the period (Fig. 4A). Then, we inoculated the cultured yeast strain to the bamboo strips, which resulted in, strikingly, restored growth and development of the second instar larvae. Finally, two of the three larvae became adult males, taking additional 74 and 201 days and attaining 9.9 and 7.0 mm of elytral length, respectively.

## Discussion

In this study, we demonstrated that (i) the inner surface of the bamboo internode containing a larva of *D. bucculenta* is always covered with a white fungal layer (Fig. 1D), (ii) a specific yeast species, *W. anomalus* ( = *P. anomala*), is repeatedly isolated from the inner wall and also from the larva (Fig. 1F and G; Fig. 2), (iii) adult females of *D. bucculenta* possess an ovipositor-associated exoskeltal pocket on the tergum of the eighth abdominal segment (Fig. 3), (iv) the putative mycangium contains yeast-like particles (Fig. 3D), (v) PCR and sequencing of a fungal gene fragment identify the mycangium-associated yeast as *W. anomalus* (Fig. 2), (vi) larvae of *D. bucculenta* neither grow nor develop in the absence of *W. anomalus* (Fig. 4A and B), and (vii) larvae of *D. bucculenta* can normally grow, develop, pupate and become adult when cultured *W. anomalus* is provided as food (Fig. 4A and C). Based on these results, we conclude that *D. bucculenta* is in an obligate symbiotic association with *W. anomalus*. To our knowledge, this study is the first to report a symbiotic association with specific microorganisms in lizard beetles (Coleoptera: Erotylidae: Languriinae).

From distant geographic populations (Miyazaki and Ibaraki, Japan) and different isolation sources and developmental stages (bamboo inner surface, larval body surface and adult female mycangium), genetically indistinguishable strains of *W. anomalus* were consistently detected (Fig. 2), which indicate an intimate and stable (at least to some extent) association between *D. bucculenta* and *W. anomalus*. Here it should be noted that, since 26S rRNA and EF-1α genes are conservative and slow-evolving genes, some unrecognized genetic variations may exist among the fungal strains. It seems plausible that the female mycangium plays an important role for sustaining the host-symbiont association by mediating vertical transmission of the symbiont upon oviposition, although the exact transmission process and involvement of the mycangium require experimental verification. Judging from the anatomy of the female ovipositor (Fig. 3D), the yeast cells contained in the mycangium will be exposed to outside when the ovipositor is stretched for oviposition. It deserves future studies how and when the yeast cells are incorporated into the mycangium, whether *W. anomalus* is selectively incorporated and cultured in the symbiotic organ, and how the yeast cells are inoculated to the bamboo internode upon oviposition.

In a number of non-social beetles and other insects, although external exoskeletal pits, pockets and cavities have often been recognized and naively referred to as “mycangia”, their fungal storage/transport/inoculation roles have been rarely verified [20], with a few exceptions such as lymexylid beetles [10], wood wasps [17], gall midges [18], leaf-rolling weevils [14] and stag beetles [19]. The similarity in the mycangial configuration, namely fungus-carrying pouch(es) associated with female ovipositor, among the lymexylid beetles, stag beetles and *D. bucculenta* seems notable, plausibly reflecting their common habit of inoculating their microbial associates to woody substrate upon oviposition. The case of *D. bucculenta* is remarkable in that a specific mycangium-associated fungus is unequivocally identified and, furthermore, an essential biological role of the fungus for the host is experimentally demonstrated.

Mueller et al. [1] pointed out that the highest levels of cultivation mutualism as those found in ants, termites and ambrosia beetles entail the following features: (a) “habitual planting” or “inoculation” of specific fungal associates to appropriate substrates; (b) “conditioning” of the fungal associates by improving their growth and proliferation and/or “protection” of the fungal associates against pests/parasites/pathogens by monitoring, sequestration and/or antibiotic application; (c) “harvesting and consumption” of the fungal associates for food; and (d) “obligate nutritional dependence” on the fungal associates. Notably, the *D. bucculenta*-*W. anomalus* relationship evidently exhibits the features (a) inoculation, (c) harvesting/consumption and (d) obligate nutritional dependence, which indicate a high level of cultivation mutualism in this non-social beetle. Confirmation of the feature (b) conditioning/protection requires further studies, although the substantial monoculture of *W. anomalus* in larva-inhabiting bamboo internodes and the invasion of non-specific fungi into an adult-inhabiting bamboo internode suggest the possibility of larval conditioning of fungal growth and/or larval control over microbial contaminants in *D. bucculenta*. Alternatively, the fungus itself may be responsible for its own monoculture in the bamboo internodes, on the ground that *W. anomalus* ( = *P. anomala*) has often been referred to as “killer yeast” or “biocontrol yeast” on account of its inhibitory effects against molds and bacteria [38]–[42]. The inside of bamboo internode is a tightly packaged, firmly isolated, and substantially sterile ecological niche, and it is difficult to utilize for most animals. By evolving the exaggerated mandibles for biting the bamboo culm and the elongated forelegs for holding itself on the bamboo surface (Fig. 1A), *D. bucculenta* succeeded in exploitation of this untouched ecological niche that is suitable for monoculture of the fungal associate.

The relationship between *D. bucculenta* and *W. anomalus* seems certainly mutualistic, but the extents of their mutual interdependence are likely asymmetric. Since *D. bucculenta* cannot grow without the fungal symbiont (Fig. 4), the host is obligatorily dependent on the symbiont. On the other hand, although *W. anomalus* may benefit from the monoculturing and vectoring activities of *D. bucculenta*, the yeast can grow independently of the insect on standard microbiological media (Fig. 1F), suggesting that the symbiont is dependent on the host not obligatorily but only facultatively. It seems likely, although experimental confirmation is needed, that the fungal symbiont has both symbiotic and free-living life stages, like some fungal cultivars of leaf-cutting ants and ambrosia beetles [3], [25], potentially being subjected not only to vertical transmission but also to horizontal transmission across host insect generations. In many host-symbiont mutualisms among insects such as aphid-*Buchnera* and tsetse-*Wigglesworthia* associations, the symbionts are unable to survive outside their hosts, are subjected to strict vertical transmission through host generations, and often co-speciate with their hosts over evolutionary time [43], [44]. In host-symbiont mutualisms among many marine invertebrates and plants, and also in some insects, by contrast, the symbionts are freely present in the environment, the hosts acquire their symbionts from the environment every generation, and the host-symbiont relationship is consequently promiscuous both ecologically and phylogenetically [45], [46]. Probably the *D. bucculenta*-*W. anomalus* relationship represents an intermediate between these typical extremes of host-symbiont mutualism.

For a long time, lizard beetles were placed in the family Languriidae, but recent phylogenetic studies have revised the taxonomic treatment of the group as the subfamily Languriinae within the family Erotylidae, whose members, pleasing fungus beetles, are mostly fungivorous [47], [48]. Although ecological aspects of lizard beetles have been poorly documented, several literatures describe that members of the Languriinae are mostly phytophagous [21]–[23]. The discovery of the fungal farming on a plant substratum in *D. bucculenta*, together with its phylogenetic affinity to pleasing fungus beetles, suggests the possibility that some lizard beetles are actually not phytophagous but fungivorous.

Using light and scanning electron microscopy, van Zandt et al. [49] identified two pairs of deep pits on the ventral aspect of the gena between the eyes and the maxillae of the erotylid beetle *Loberus impressus*, and suggested their possible role as mycangia in addition to the role as glandular outlets. On the other hand, Grebennikov and Leschen [20] pointed out that the majority of reports on mycangial function of insect exoskeletal cavities are based solely on the observation of these structures and the fungus-associated ecology of the insects, usually without other biological evidence. Hence, whether the exoskeletal pits of *L. impressus* comprise mycangia is currently elusive. It is evident that the ovipositor-associated mycangium of *D. bucculenta* is structurally, developmentally and evolutionarily unrelated to the external pits on the head of the erotylid beetle.

In conclusion, we discovered a fungal cultivation mutualism in a non-social beetle *D. bucculenta*, which entails association with a specific yeast species, development of a mycangium adjacent to the female ovipositor, and obligate nutritional dependence on the fungal associate. The high level of cultivation mutualism in the non-social insect would provide a valuable insight into the evolutionary trajectories toward the highest levels of cultivation mutualisms, so-called agriculture, found in social insects, and ultimately, human beings.
